# Clinical Efficacy and Safety of Different Dental Prosthetic Membranes in Guided Bone Regeneration during Dental Implants: A Meta-Analysis

**DOI:** 10.1155/2022/3245014

**Published:** 2022-07-31

**Authors:** Yan Guo, Linghan Su, Caidi Chen, Yan Liu, Jianxue Li

**Affiliations:** ^1^Department of Stomatology, The 940th Hospital of Joint Logistic Support Force of PLA, Lanzhou 730050, China; ^2^Endocrinology Department, The 940th Hospital of Joint Logistic Support Force of PLA, Lanzhou 730050, China; ^3^The 940th Hospital of Joint Logistics Support Force of PLA, Lanzhou 730050, China

## Abstract

*Objective*. To evaluate clinical efficacy and safety of absorbable and non-absorbable dental restorative membranes in guided bone regeneration (GBR). Articles concerning absorbable and non-absorbable prosthetic membrane-related studies of GBR were screened from multiple databases. In the end, 526 postoperative patients who met eligibility criteria were screened for the study from eight trials. The results showed that the repair success rate of the experimental group (absorbable dental restorative membrane) was higher than that of the control group (non-absorbable dental restorative membrane) (RR = 1.18, 95% CI [1.11,1.26], and the total physical therapy effect was *P* < 0.0001, *I*^2^ = 0%), and the height of bone graft in the experimental group was higher than that in the control group (MD = 0.67, 95% CI [0.11, 1.23]). The thickness of bone graft in the experimental group was higher than that in the control group (MD = 0.43, 95% CI [0.30,0.56], *P* < 0.00001, *I*^2^ = 61%), and the adverse events in the experimental group were less than those in the control group (RR = 0.31, 95% CI [0.18, 0.51], *P* < 0.00001, *I*^2^ = 13%). Absorbable prosthetic membrane is superior to non-absorbable prosthetic membrane in clinical efficacy and safety.

## 1. Introduction

Guided Bone Regeneration (GBR) originated from the field of periodontology in guided tissue regeneration technology. It is a biofilm made of biomaterials, which erect a biological barrier between bone defects and gingival soft tissue [1–3]. GBR prevents epithelial cells and fibroblasts in soft tissue and soft tissue from growing into the bone defect area. This process ensures that the osteogenesis process is completed on the premise of no interference of fibroblasts. Finally, GBR can realize complete bone repair of the defect area, which needs oral repair membrane [4].

Oral repair membrane is a biocompatible material. The repair membrane is placed between oral soft tissue and bone defect by surgery to establish a biological barrier to create a relatively closed bone regeneration environment [5, 6]. Oral repair film can be divided into absorbable film and non-absorbable film according to whether the material can be degraded. In the past, patients used titanium membrane (non-absorbable membrane) as a protective barrier membrane because titanium membrane could not be fully absorbed. This process limited the supply of blood plasma and then hindered the blood supply in the bone graft area, which had a significant impact on the recovery of patients to a certain extent [7–9]. However, it has good plasticity and can bend, trim the contour, adapt to various bone defect forms, better stabilize the wound, and guide bone regeneration [10].

Although the absorbable membrane risks rapid degradation, it makes the new bone tissue adhere to the biofilm. Absorbable membrane promotes the early tissue integration and the production of transmembrane blood vessels, avoids the inward growth of connective tissue, increases the stability of gingival tissue, and reduces gingival atrophy [11]. Also, it can reduce patient complications without the need for second-stage surgical removal of the membrane.

Although there are several research studies about comparison between absorbable and non-absorbable dental restorative membrane in guided bone regeneration, there is little comprehensive analysis for the topic. Therefore, we conducted this research to overall analyze the difference in absorbable and non-absorbable dental restorative membrane in guided bone regeneration.

In this paper, we have evaluated clinical efficacy and safety of absorbable and non-absorbable dental restorative membranes in guided bone regeneration (GBR). For this purpose, both absorbable and non-absorbable prosthetic films for GBR were selected from multiple databases (PubMed, Web of Science, Cochrane Library, and China National Knowledge Infrastructure), whereas Review Manager 5.2 was used for meta-analysis, sensitivity analysis, and bias analysis. After the screening process, 526 postoperative patients were extracted from 8 trials which are those patients who finally met the qualification criteria to conduct this meta-analysis.

## 2. Proposed Method or Strategy

To ensure the scientificity, we followed PRISMA statement and the methods of Cao et al. [12].

### 2.1. Literature Search Strategy

We have searched the randomized controlled trials published by PubMed, ScienceNet, Cochrane Library, and China National knowledge Infrastructure from January 1, 2000, to September 1, 2021, using the following search terms:

1. Absorbable dental repair membrane.
2. Bone regeneration.
3. Clinical effect. The search strategy involves medical subject headings (mesh) and text words combined by the Boolean operator “AND.”

We will conduct a comprehensive search in multiple databases without restrictions on language or publication status. In order to maximize the specificity and sensitivity of the search, the author should also refer to the list of retrieved references to find other relevant studies not found through the search strategy.

A comprehensive review of potentially relevant articles was conducted to ensure that they met all inclusion criteria, as follows:

1. Studies comparing patients receiving absorbable and non-absorbable dental repair membranes.
2. Studies comparing patients receiving absorbable and non-absorbable dental repair membranes.
3. GBR patients.
4. Between absorbable and non-absorbable dental restorative membranes, indexes for evaluating curative effect or other relevant indexes are included.
5. The full text is available for reference.

Studies were excluded according to the following predetermined exclusion criteria:

1. Studies on other subjects.
2. Comparison of other interventions.
3. Lack of research on available data.
4. Comments, abstracts, and reproduction of publications.

### 2.2. Data Extraction and Quality Assessment

Two pairs of reviewers independently screened the titles, abstracts, and full-text articles of potentially qualified studies and resolved their differences through discussion. The following data parameters were extracted: name of main author, study country, patient population in the study, number of participants in each group, patient age, patient gender, characteristics of drug intervention during follow-up in each group, and outcome measurement in each group. The Cochrane bias risk tool in Review Manager 5.2 was used to evaluate the effectiveness of qualified randomized controlled trials. Egger's test and funnel plot program were used to assess the risk of bias in the study.

### 2.3. Statistical Analysis

Review Manager (version 5.2, Cochrane Collaboration, 2011) is used to evaluate the impact of the results in the selected report. In order to measure the consistency of effect size (or and MD), DerSimonian and Laird random effect models were used for paired meta-analysis, and the combined estimates and 95% CI between two groups were calcultated. 0% to 40% of heterogeneity is considered “may not be important,” 30% to 60% is considered “moderate heterogeneity,” 50% to 90% is considered “substantial heterogeneity,” and 75% to 100% is considered “considerable heterogeneity.”

If *P* < 0.05 or *I*^2^ > 50%, the random effect model was used for analysis; if *P* ≥ 0.05 and *I*^2^ ≤ 50%, the fixed effect model was used for analysis. When heterogeneity exists, the random effect model is used, while the fixed effect model is applied. Publication bias was examined by visual examination of the funnel plot and using Egger's test. Sensitivity analysis was performed by deleting one study at a time to observe the impact of individual results on the overall analysis.

## 3. Results

### 3.1. Search Process

The initial search yielded 966 articles from four databases, including PubMed, Embase, Web of Science, and CNKI. After the first screening, 880 records were retained. By screening titles and abstracts, additional 823 records were excluded because they were review articles, letters, case reports, comments, or editorials. Then, 57 articles were remained. Eight articles were further excluded for various reasons, including different research designs or insufficient available data.

**Figure 1:**
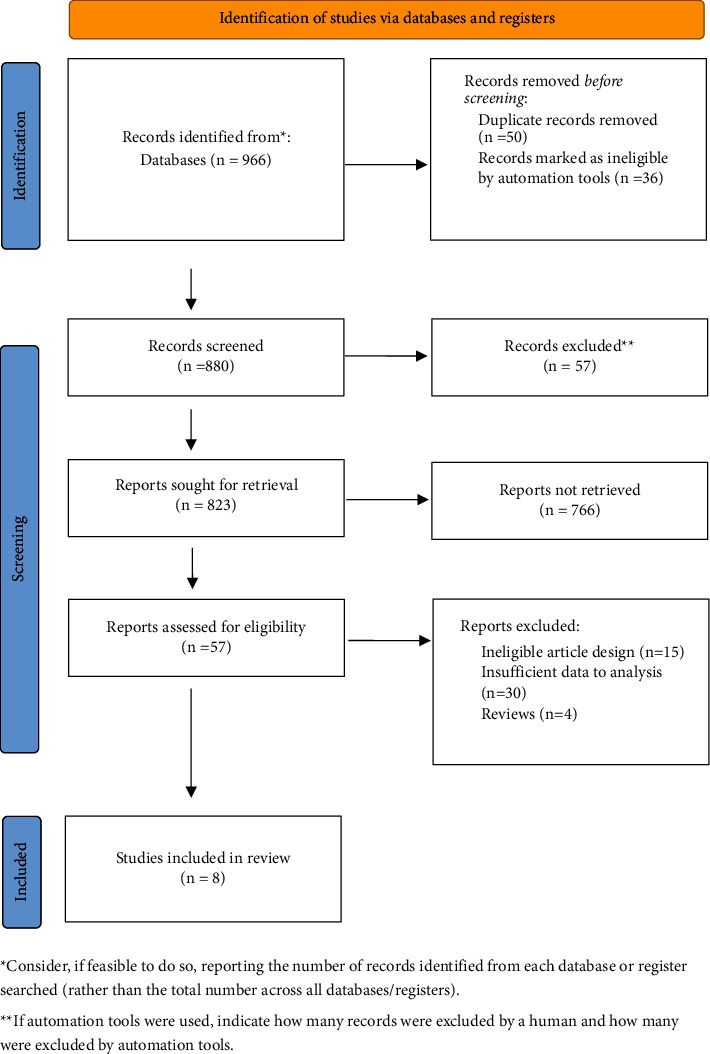
PRISMA flowchart detailing the search strategy for study inclusion.

Finally, 8 studies [13–20] met the inclusion criteria and were included in this meta-analysis, with a total of 526 patients. The process followed PRISMA guidelines, including the reasons for excluding the study, as shown in Figure 1.

### 3.2. Characteristics of Included Studies

Table 1 lists the main characteristics of the eight tests. These studies included 526 patients (263 patients in the experimental group and 263 patients in the control group). All 8 articles were published from 2016 to 2020. The sample size is between 26 and 100.

### 3.3. Results of Quality Assessment

**Figure 2:**
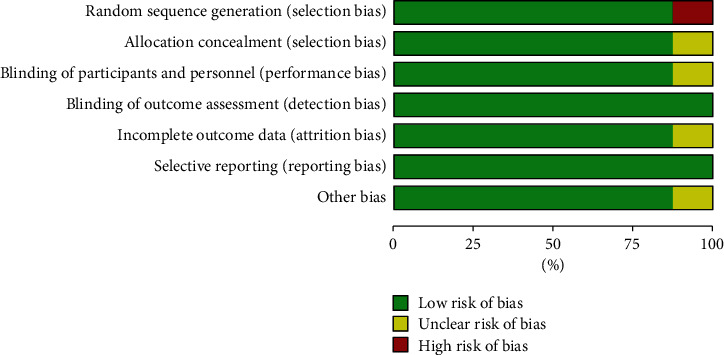
Graph of the risk of bias: green = low risk; yellow with question mark = unclear; and red = high risk.

**Figure 3:**
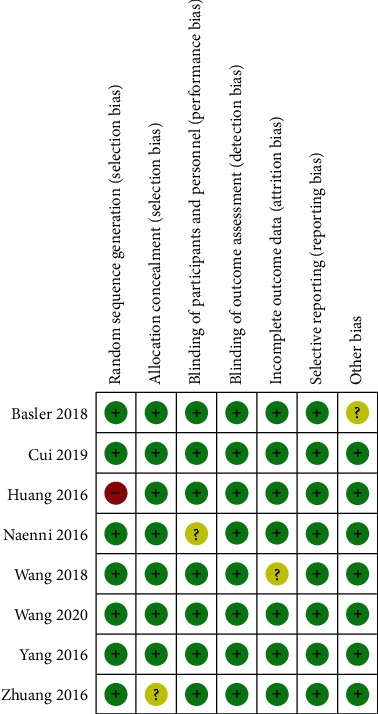
Risk of bias for each study, using three colors: green = low risk; yellow with question mark = unclear; and red = high risk.

The Cochrane bias risk assessment tool was used to assess the risk of inclusion in the study. Of these 8 articles, only 1 study found a high risk of selection bias, performance bias, detection bias, abrasion bias, reporting bias, and other bias (Figures 2 and 3).

Given the deviation summary, only 1 clue has different deviation. Visual examination of the funnel chart of studies reporting efficiency showed some asymmetry, and the Egger test showed little evidence of publication bias.

### 3.4. Results of Heterogeneity Test

#### 3.4.1. Heterogeneity Analysis of Successful Repair between Experiment and Control Groups

**Figure 4:**
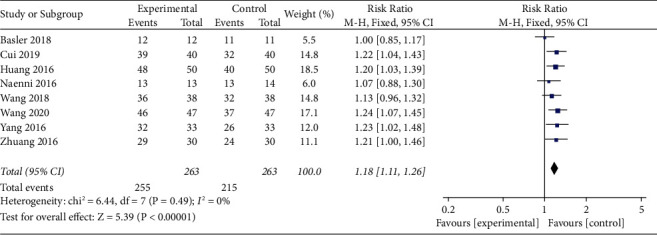
Forest plots for the effects for successful repair in experiment versus control groups.

Meta-analysis of successful repair. The overall results showed that the repair success rate of the experimental group was higher than that of the control group (RR = 1.18, 95% confidence interval [1.11, 1.26], and the total effect was *P* < 0.0001, *I*^2^ = 0% fixed effect model) (Figure 4).

#### 3.4.2. Heterogeneity Analysis of Height of Bone Graft between Experiment and Control Groups

**Figure 5:**
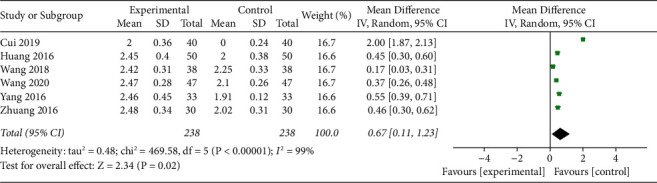
Forest plots for the height of bone graft in experiment versus control groups.

Similarly, the first micturition (min) between the experimental group and the control group was meta-analyzed. The overall results showed that the height of bone transplantation in the experimental group was higher than that in the control group (MD = 0.67, 95% confidence interval [0.11, 1.23], overall *P* = 0.02, *I*^2^ = 99%, using random effect model) (Figure 5).

#### 3.4.3. Heterogeneity Analysis of Bone Graft Thickness between Experiment and Control Groups

**Figure 6:**
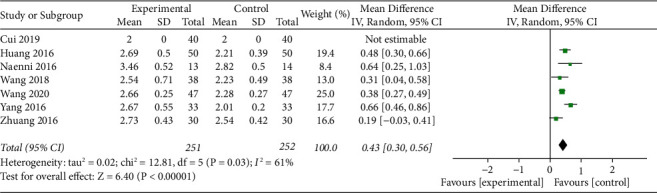
Forest plots for bone graft thickness in experiment versus control groups.

For residual urine, it was reported in 7 studies. The overall results showed that the thickness of bone graft in the experimental group was higher than that in the control group (MD = 0.43, 95% confidence interval [0.30, 0.56], *P* < 0.00001, *I*^2^ = 61%, using random effect model) (Figure 6).

#### 3.4.4. Heterogeneity Analysis of Adverse Events between Experiment and Control Groups

**Figure 7:**
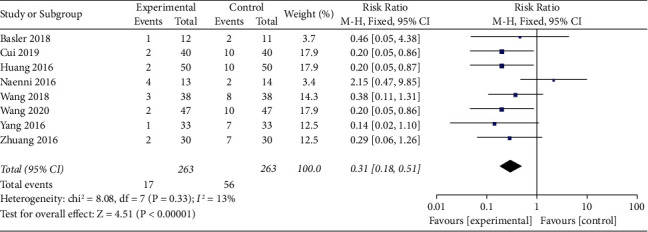
Forest plots for adverse events in experiment versus control groups.

To better assess the safety of different therapies, we collected data on adverse events. The overall results showed that the adverse events in the experimental group were less than those in the control group (RR = 0.31, 95% confidence interval [0.18, 0.51], *P* < 0.00001, *I*^2^ = 13%, using the fixed effect model) (Figure 7).

### 3.5. Results of Sensitivity Analysis and Publication Bias

**Figure 8:**
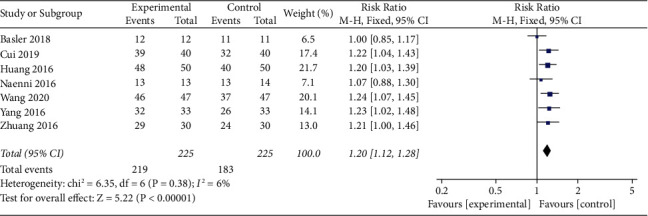
Sensitivity analysis of effects for successful repair between experiment and control groups.

To assess the sensitivity of the articles, we deleted a study to observe the effect of individual outcomes on the overall efficacy of urinary retention. In Figure 4, the result shows *I*^2^ = 0% high heterogeneity. When Wang's article [18] was deleted, the results change the most, indicating the robustness of the included study (Figure 8).

**Figure 9:**
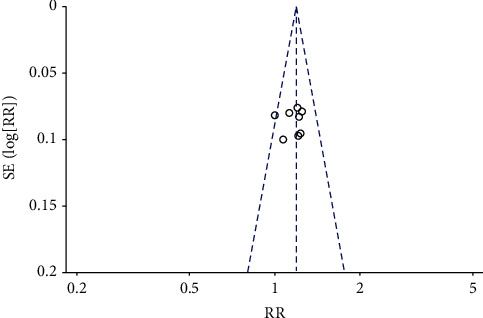
Funnel plot of publication bias.

We used funnel plots to assess the efficiency of urinary retention. Visual results showed symmetrical shape. The *P* value of Egger test was 0.218, indicating that there was no publication bias in this study (Figure 9).

## 4. Discussion

From our results, we can find that absorbable dental restorative membrane had higher successful repair than non-absorbable dental restorative membrane in guided bone regeneration. In addition, height of bone graft and bone graft thickness were both higher in absorbable dental restorative membrane than non-absorbable dental restorative membrane. In the comparison of safety, absorbable dental restorative membrane was worse than non-absorbable dental restorative membrane. These results showed that absorbable dental restorative membrane was better than non-absorbable dental restorative membrane in clinical effects and safety. These were consistent with Zhang's study [21] that bone regeneration guided by absorbable biofilm in patients with GBR improves the success rate of dental implantation and has high safety.

With the development of dental implants, implant denture has become one of the conventional treatment methods for repairing dentition defects or deletions, and the methods are constantly simplified [22, 23]. The safety and reliability are gradually improved. Guided bone regeneration (GBR) is often widely used to treat periodontal diseases and repair maxillary sinus defects and bone defects. Oral repair membrane has the characteristics of high efficiency, short time consumption, thick osteogenesis, and high osteogenesis [24]. The primary function of the oral repair membrane is to prevent epithelial cells and connective tissue cells from entering the regeneration area and create and maintain a space for the unrestrained growth of pluripotent stem cells and osteoblasts [25]. It is widely used in stomatology, such as periodontal mucosa, oral implant, and alveolar surgery.

Non-absorbable membrane (titanium membrane) was a commonly used oral repair material in the past. It has the characteristics of stable space, good resistance strength, and hard texture [26]. It plays a specific role in promoting the growth of bone grafts. However, it also has some adverse effects, such as preventing the excellent absorption of blood by bone graft, prolonging patients' recovery time, and requiring secondary surgery [27]. The incidence of postoperative complications is high, and the osteogenic effect is poor.

The absorbable membrane has collagen composition similar to periodontal connective tissue, including weak immunogenicity and cytotoxicity [28]. The absorbable membrane can promote the chemotaxis of periodontal ligament (PDL) cells and gingival fibroblasts. In addition, it can encourage hemostasis, is easy to operate, and degrades physiologically. Calcification and ossification can occur when approaching bone [29].

## 5. Conclusion

In this paper, we have evaluated clinical efficacy and safety of absorbable and non-absorbable dental restorative membranes in guided bone regeneration (GBR). For this purpose, both absorbable and non-absorbable prosthetic films for GBR were selected from multiple databases (PubMed, Web of Science, Cochrane Library, and China National Knowledge Infrastructure), whereas Review Manager 5.2 was used for meta-analysis, sensitivity analysis, and bias analysis. After the screening process, 526 postoperative patients were extracted from 8 trials which are those patients who finally met the qualification criteria. The present study showed that absorbable dental restorative membrane was better than non-absorbable dental membrane both in clinical effects and safety. However, our findings should be carefully considered with caution due to small sample size. Studies in various areas with large study population are essential to further confirm our findings in the future. There are some limitations in this study. Firstly, more indicators should be included, and this could be conducted in the future. Secondly, more research studies from various areas could be analyzed in the next research.

In future, we are keen to extend the proposed study to other domains and diseases preferably in smart healthcare sector.

## Figures and Tables

**Table 1:** Characteristics of eligible studies.

Study	Year	Country	Groups	Sex (male/female)	Age (years)	*n*	Years of onset
Basler	2018	Switzerland	Resorbable membrane	11/12	56.6 ± 17.4	12	January 2015 to January 2018
			Non-resorbable membrane			11	
Cui	2019	China	Resorbable membrane	44/36	47.4 ± 4.25	40	February 2016 to February 2018
			Non-resorbable membrane			40	
Huang	2016	China	Resorbable membrane	63/37	50.8 ± 1.9	50	August 2013 to February 2015
			Non-resorbable membrane			50	
Naenni	2016	Switzerland	Resorbable membrane	13/14	51.85 ± 29.7	13	March 2010 and January 2013
			Non-resorbable membrane			14	
Wang	2018	China	Resorbable membrane	39/37	40.25 ± 18.75	38	July 2017 to July 2018
			Non-resorbable membrane			38	
Wang	2020	China	Resorbable membrane	49/45	42.4 ± 5.55	47	December 2017 to December 2018
			Non-resorbable membrane			47	
Yang	2016	China	Resorbable membrane	41/25	45.07 ± 6.5	33	January 2014 to January 2015
			Non-resorbable membrane			33	
Zhuang	2016	China	Resorbable membrane	36/24±	36.4 ± 9.09	30	January 2013 to May 2015
			Non-resorbable membrane			30	

## Data Availability

The datasets used and analyzed during the current study are available from the corresponding author upon reasonable request.

## References

[1] Monteiro D., Kim B. S. (2005). Utilizing anorganic bovine bone (bio-oss) with absorbable and nonabsorbable membranes placed over the lateral window: histomorphometric and clinical. *International Journal of Periodontics Restorative Dent*.

[2] Khoury G., Lahoud P., Younes R. (2015). *Use of Grafting Materials in Sinus Floor Elevation: Biologic Basis and Current Updates*.

[3] Anguelov B. J. (2003). Trabeculectomy with absorbable and nonabsorbable sutures of the scleral flap in primary open angle glaucoma. *国际眼科杂志*.

[4] Yong X. (2015). *The Clinical Effect Of Oral Biofilm In Guiding Of Bone Regeneration During Dental Implant*.

[5] Jiao X. F., Stomatology D. O. (2017). Study on the clinical effect of oral prosthetic membrane material guided bone regeneration in dental implantation. *China Medical Cosmetology*.

[6] Rocchietta I., Schupbach P., Ghezzi C., Maschera M. (2012). Soft tissue integration of a porcine collagen membrane: an experimental study in pigs. *The International Journal of Periodontics and Restorative Dentistry*.

[7] Wallace S. S., Froum S. J., Cho S. C. (2005). Sinus augmentation utilizing anorganic bovine bone (Bio-Oss) with absorbable and nonabsorbable membranes placed over the lateral window: histomorphometric and clinical analyses. *The International Journal of Periodontics and Restorative Dentistry*.

[8] Pfoerringer D., Harrasser N., Muehlhofer H. (2018). Osteoinduction and -conduction through absorbable bone substitute materials based on calcium sulfate: in vivo biological behavior in a rabbit model. *Journal of Materials Science*.

[9] Lopez M., Olate S., Lanata-Flores A. L. (2013). New bone formation in a bone defect associated to dental implant using absorbable or non-absorbable membrane in a dog model. *International Journal of Clinical and Experimental Pathology*.

[10] Sallum E. A., Sallum A. W., Nociti F. H., Marcantoniode R. A., Toledo S. D. (1998). New attachment achieved by guided tissue regeneration using a bioresorbable polylactic acid membrane in dogs. *The International Journal of Periodontics and Restorative Dentistry*.

[11] Joop A., Rahlf B., Gellrich N. C., Kampmann A., See C. V., Stoetzer M. (2017). Examination of local periosteal microcirculation after application of absorbable and non-absorbable membranes. *Journal of Oral Implantology*.

[12] Cao M., Wu X., Xu J. (2022). A systematic review and meta-analysis of neostigmine for urinary retention after surgeries. *Translational Andrology and Urology*.

[13] Zy C. (2019). Effects of two different oral repair membrane materials on implant guided bone regeneration. *Grassroots Medical Forum*.

[14] Hb H. (2016). Clinical study on the effect of dental restorative membrane in guided bone regeneration during dental implantation. *Shenzhen Journal of integrated traditional Chinese and Western Medicine*.

[15] Zhou L. (2016). Evaluation of the effect of dental restorative membrane material in guiding osteogenesis during dental implantation. *Heilongjiang Medicine Journal*.

[16] Hj W. (2020). Application of Haiao oral repair membrane in guided bone regeneration during dental implantation and analysis of its adverse reactions. *Modern diagnosis and treatment*.

[17] Qy Y. (2016). Comparative study on guided bone regeneration of different oral restorative membrane materials in dental implantation. *Chin J Mod Drug Appl*.

[18] Qx C. P. (2018). Clinical effect of different oral restorative membrane materials on guided bone regeneration during dental implantation. *Medical Information*.

[19] Basler T., Naenni N., Schneider D., Hammerle C. H. F., Jung R. E., Thoma D. S. (2018). Randomized controlled clinical study assessing two membranes for guided bone regeneration of peri-implant bone defects: 3-year results. *Clinical Oral Implants Research*.

[20] Naenni N., Schneider D., Jung R. E., Husler J., Hammerle C. H. F., Thoma D. S. (2017). Randomized clinical study assessing two membranes for guided bone regeneration of peri-implant bone defects: clinical and histological outcomes at 6 months. *Clinical Oral Implants Research*.

[21] Zhang J. (2018). Application and effect of absorbable repair membrane in guided bone regeneration of dental implant. *Chinese Community Doctors*.

[22] Battistella E., Varoni E., Cochis A., Palazzo B., Rimondini L. (2011). Degradable polymers may improve dental practice. *Journal of Applied Biomaterials and Biomechanics*.

[23] Sakallioglu U., Yavuz U., Lütfioglu M., Keskiner I., Açikgöz G. (2007). Clinical outcomes of guided tissue regeneration with Atrisorb membrane in the treatment of intrabony defects: a 3-year follow-up study. *The International Journal of Periodontics and Restorative Dentistry*.

[24] Rodriguez P. A., Lenarduzzi A. L., Amer M., Sierra L. Bone-regeneration after Apical Surgery Using Reabsorbable Membrane and Powder Bone.

[25] Cao T., Ohno K., Shirota T., Michi K. (2012). Bone defect healing by nonabsorbable membrane for guided tissue regeneration. *Dental Medicine Research*.

[26] Medel S., Alarab M., Kufaishi H., Drutz H., Shynlova O. (2015). Attachment of primary vaginal fibroblasts to absorbable and nonabsorbable implant materials coated with platelet-rich plasma. *Female Pelvic Medicine & Reconstructive Surgery*.

[27] Shao B., Lu Y., Sui J. (2016). Application of dental restorative materials in bone regeneration in dental implantation. *World Latest Medicine Information*.

[28] Nociti F. H., Machado M. Â N., Stefani C. M., Sallum E. A., Sallum A. W. (2001). Absorbable versus nonabsorbable membranes and bone grafts in the treatment of ligature-induced peri-implantitis defects in dogs. *Clinical Oral Implants Research*.

[29] Froum S. J., Froum S. H., Rosen P. S. (2015). A regenerative approach to the successful treatment of peri-implantitis: a consecutive series of 170 implants in 100 patients with 2- to 10-year follow-up. *The International Journal of Periodontics and Restorative Dentistry*.

